# Exposure to Heated Tobacco Products and Adverse Health Effects, a Systematic Review

**DOI:** 10.3390/ijerph18126651

**Published:** 2021-06-21

**Authors:** Małgorzata Znyk, Joanna Jurewicz, Dorota Kaleta

**Affiliations:** Department of Hygiene and Epidemiology, Medical University of Lodz, 90-647 Lodz, Poland; joanna.jurewicz@umed.lodz.pl (J.J.); dkaleta@op.pl (D.K.)

**Keywords:** heated tobacco products, heat-non-burn, IQOS, Glo, Ploom, adverse health effects

## Abstract

Heated tobacco products (HTP) are a form of nicotine delivery intended to be an alternative to traditional cigarettes. HTP tobacco products are sold to consumers as a less harmful alternative to traditional cigarettes, both for users and bystanders. The actual impact of HTP on the health of users and its overall impact on public health is still not fully known. A systematic search of the literature was carried out to identify relevant studies published in English from 2015 to February 2021. The following databases were used: PubMed, Scopus, Elsevier and ClinicalKey. 25 studies (independent and sponsored by the tobacco industry) were considered. The analysis of exposure biomarkers and cardiovascular and respiratory biomarkers showed differences between smokers and people using heated tobacco products. Improvements in clinically relevant risk markers, especially cholesterol, sICAM-1, 8-epi-PGF2α, 11-DTX-B2, HDL and FEV1, were observed compared to persistent cigarette smokers. On the other hand, exposure to IQOS has been reported to alter mitochondrial function, which may further exaggerate airway inflammation, airway remodeling and lung cancer. These products have the potential to increase oxidative stress and increase respiratory tract infections by increasing microbial adherence to the respiratory tract. Our review suggests that HTP products may be products with a reduced risk of chronic diseases, including respiratory and cardiovascular diseases and cancer compared to traditional smoking, although in the case of non-smokers so far, they may pose a risk of their occurrence. Research seems to be necessary to assess the frequency of HTP use and its potential negative health effects.

## 1. Introduction

Heated tobacco products (HTP) are a form of nicotine delivery that is to be an alternative to traditional cigarettes [1]. HTP tobacco products are sold to consumers as a less harmful alternative to traditional cigarettes, both for users and for bystanders [2], though the HTP tobacco aerosol contains various harmful components, albeit in lower amounts than smoke from combustible materials [3,4].

The tested heated tobacco products delivered more nicotine in aerosol than a Cigalike e-cigarette, and less nicotine than a tank-style e-cigarette. The limited evidence on environmental emissions from the use of heated tobacco products suggests that harmful exposure from heated tobacco products is higher than from e-cigarettes, but further evidence is needed to be able to compare products [5].

Several types of HTP have been introduced to the market so far, including IQOS from Philip Morris International (PMI), Glo from British American Tobacco, Ploom from Japan Tobacco International, Pax products from Pax Lab and iFuse [6]. In 2013, Tobacco International (JTI) introduced Ploom in Japan. In 2014, Philip Morris International (PMI) launched IQOS. In 2015, British American Tobacco (BAT) launched iFuse for the first time in Romania. BAT then sold Glo in Asia [7]. Currently, HTP is sold in about 40 countries, and IQOS is found in most of them.

Tobacco heating technology is based on a unique electronic heating method to produce aerosols from tobacco stakes. Tobacco heating systems operate at lower temperatures (240–350 °C) than traditional cigarettes (>600 °C) [1], which reduced use temperature, and allows the aerosol to be produced without burning tobacco [8].

About 3.1 million people use IQOS in Japan, while 4.0 million use any HNB or e-cigarette, and 2.9 million are dual users [9]. In a nationwide adult Japanese survey (2015), 48% were aware of HTP, with 7% reporting having used the products before and 1.3% reporting use in the last 30 days [10]. In 2015, 0.3% of the Japanese population aged 15–69 reported using IQOS in the last 30 days, while in 2017 it was already 3.6% [10]. In 2017, 4.7% of Japanese respondents used at least one type of e-cigarette or HNB tobacco; of these, 72% now also smoked flammable cigarettes, representing 3.4% of the overall dual-use rate [9]. 9.3% of the adult UK population was aware of the existence of HnB, and 1.7% have tried or used these products. Among people who have ever tried HnB, 39% tried it once or twice, and 13% every day [11]. In 2017, 1.4% of the Italian population aged ≥ 15 years had tried IQOS. Overall, 1.0% of non-smokers, 0.8% of ex-smokers and 3.1% of current cigarette smokers have tried IQOS [12]. In the UK, 1.7% of adults have tried or used HTP, but only 13% used it daily in 2017 [11]. In the United States, about one in 20 American adults was aware of the use of HnB tobacco products, including one in ten who are now a traditional smoker [13]. Total sales for HTP in 2016 were US$2.1 billion, and they are expected to reach US$17.9 billion by 2021 [6]. Organizations and governments concerned with tobacco control should closely monitor HNB (a heat-non-burn) tobacco and consider how to regulate it [9]. The actual impact of HTP on the health of users and its overall impact on public health are still not fully known [14].

The data shows that HTP is mainly used in combination with other products, mainly by the youngest generation and those who never smoke. The dual use of these products, their high use among younger generations and the interest of non-smokers are worrying, and point to the need for close monitoring of HTP for prevalence and user characteristics [15]. Only a few independent studies, mainly from Japan, where HTP are widespread, and the United States have investigated their spread and/or public health consequences [1,9,10,16].

Despite the lack of convincing evidence regarding the harmful effects of HTP, growing evidence indicates that the IQOS tobacco heating system may not be as safe as the manufacturer claims; this is due to its potentially harmful ingredients and the increased concentration of nicotine and other harmful chemicals emitted by HTP devices [4,8,17].

According to the tobacco industry data, aerosols generated during the heating process have approximately 90–95% lower levels of toxic substances than conventional cigarette smoke [18,19,20,21]. Independent studies have confirmed that the concentration of chemical compounds produced by HTP is lower than that produced by traditional cigarettes [22,23,24]. However, toxic compounds were not completely removed from the heated tobacco aerosol [4,20,25,26,27,28,29,30]. Many studies, both independent and industry, have shown that IQOS sticks contained 70–80% of the concentration of nicotine found in traditional cigarettes [3,4,20,22,24,31]. Farsalinos et al. reported that HTPs deliver nicotine to the aerosol at levels higher than e-cigarettes [31]. Independent studies have shown that IQOS emit significantly lower levels of carbonyls and submicron particles than commercial cigarettes, but higher levels than e-cigarettes [27,28]. Mallock et al. observed significantly lower levels of aldehydes (approx. 80–95%) and volatile organic compounds (approx. 97–99%) in the IQOS aerosol, compared to cigarette smoke [24]. Pacitto et al. also showed lower concentrations of volatile and non-volatile particles in IQOS aerosols compared to traditional cigarette smoke [29]. Ruprecht et al. noticed that the IQOS aerosol was free of metal emissions, unlike the aerosols of cigarettes and e-cigarettes [30]. In the study performed by Rodrigo et al., 2021 non-cancer and cancer risks were estimated in a range of HTP and commercial combustible cigarettes, based upon their harmful and potentially harmful constituent yields in aerosols and smoke. It was found that mean lifetime cancer risk values were decreased by more than one order of magnitude when comparing HTPs and commercial cigarettes. Additionally, the higher margin of exposure for non-cancer risk was observed for HTPs when compared to commercial cigarettes [32]. Due to conflicting results and the limited knowledge of IQOS exposure, we performed the review to analyse the potential effect of heated tobacco products on human health.

## 2. Materials and Methods

The PRISMA [33] (preferred reporting items for systematic reviews and meta-analyses) was employed to guide this review.

### 2.1. Search Strategy

A systematic search of the literature was carried out to identify relevant studies published in English from 2015 to February 2021. The most recent literature from the last six years is included. The period was chosen to reflect findings over the past six years, during which IQOS started to be used more frequently, and there were few studies conducted on the effect of exposure to HTP prior to 2015 (in 2013, the Japan Tobacco International introduced Ploom, and in 2014 Philip Morris launched IQOS). At that time, the availability of sensitive, specific and affordable bioassays made biomarkers feasible for use in epidemiological studies for measuring exposures to those compounds. Additionally, during this time, growing rodent literature provided convincing data on the adverse effect of exposure to heated tobacco products.

The following databases were used: PubMed; Scopus; Elsevier; and ClinicalKey. The keywords for our search included a combination of terms related to the potential effect of using the heated tobacco product. We identified relevant records using the following search terms: heated tobacco products and adverse health effects, IQOS and Glo and Ploom, and health effects.

In total, 502 articles were found as a result of the search, and they were all checked for eligibility.

The reference lists of the selected articles were subject to a hand search to identify additional articles.

### 2.2. Selection Criteria

25 publications on adverse health effects after exposure to heated tobacco products were selected by two reviewers, with an excellent agreement (k = 0.80). Independent and sponsored studies by the tobacco industry were included.

Inclusion criteria: the review included peer-reviewed studies that looked at exposure to heated tobacco products and whether there was an association between heated tobacco products and adverse health effects in humans. In vitro studies were also included. The majority of articles (80%) did not meet the inclusion criteria for our study, as they did not address the health effects of HTP use.

Exclusion criteria: the publication contains the same data as the previous publication; published before 2015, and the publication in different language than English.

### 2.3. Study Selection

Two researchers identified relevant articles, and independently assessed which ones should be included in this review, and incongruences were resolved by discussion and the intervention of a third independent author. Articles were displayed by title and abstract. Duplicate and irrelevant items have been excluded. The remaining articles were subject to a full-text review. The following information was taken into account when selecting the studies: authors and years of publication; the main purpose of the study; results; type of study (in vitro, epidemiological study); and accurate results. 25 studies were considered. Only original articles with assessment of the effect on both humans and in vitro were included. Finally, for the purpose of this review, from each study the following information was abstracted: study population; cell type; type of outcome (cardiovascular disease, respiratory disease); and type of exposure and methods used for its assessment (including biomarkers). Selected studies were presented in Table 1.

## 3. Results

### 3.1. The Effect of Exposure to Heated Tobacco Products on Human Health

#### 3.1.1. Respiratory System

The association between exposure to HTP and respiratory diseases was examined in dependent and independent studies [9,34,35,36,37,38,39,40,41,42,43]. Three of these studies had been conducted in Japan [9,40,41] and subsequently two in Poland, one in the US, one in the UK, one in Australia, one in Switzerland, one in Belgium and one in Korea [34,35,36,37,38,39,42,43]. Most of these studies were in vitro studies, while only five were performed in humans in Japan, Belgium and Korea [9,40,41,42,43]. Respiratory health effects were examined in three dependent and eight independent studies.

A study by Van der Toorn et al. (2018) showed that heated tobacco products can reduce the risk of lung cancer. Long-term exposure to the total particulate matter from IQOS had a lower biological impact on the human bronchial epithelial cells line compared to the total particulate matter from cigarette smoke. The study assessed the functional and molecular changes during long-term exposure of human bronchial epithelial cells to total particulate matter (TPM) from a tobacco heating system (THS) 2.2. It was shown that TPM from THS 2.2 had less biological effect than TPM from 3R4F on human bronchial epithelial cells. During long-term exposure of BEAS-2B cells to TPM from 3R4F CS and THS 2.2 aerosols, changes in epithelial morphology and phenotype were observed. Repeated exposure of BEAS-2B cells to TPM from the THS 2.2 aerosol, compared to TPM from the smoke of the reference 3R4F cigarette, induced continuous changes in gene expression, as well as phenotypic changes in cellular transformation rates [39].

Furthermore, Sohal et al. (2019) investigated whether exposure to IQOS has the same destructive effect on human respiratory epithelial cells and smooth muscle cells as traditional tobacco and in vitro e-cigarettes. The results were different than these reported by Van der Toorn et al. (2018). It was found that exposure to IQOS was as toxic as cigarette smoke extract (CSE). Moreover, exposure to IQOS showed a similar induction to CSE, suggesting that IQOS is as effective as CSE in inducing the release of chemokines from both types of airway cells [35]. This study found that exposure to IQOS weakens cell homeostasis in the human respiratory tract. The differences in results may be explained by the fact that the toxic threshold for toxicants in Sohal et al.’s 2019 study was not established. So, simple excess exposures of cells may explain the equal toxicity of the HNB versus combustible product exposure.

IQOS has the potential to increase inflammation, infection and initiate EMT-related changes in the airways of users of these devices, as has been observed in smokers [35,44]. These studies show that HTP can alter important physiological functions of the lungs. Moreover, IQOS may increase oxidative stress and the number of respiratory infections by increasing the adherence of microorganisms to the respiratory tract [37,45]. Mitochondrial dysfunction caused by cigarette smoke is involved in the pathology of respiratory diseases caused by oxidative stress. Reducing the levels of harmful and potentially harmful components by heating instead of burning tobacco can reduce mitochondrial changes that contribute to oxidative stress and cell damage [38].

A pilot study by Leigh et al. (2018) reported that HTP emissions caused damage to human bronchial epithelial cells compared to air controls. HTP showed higher cytotoxicity compared to the air controls using the neutral red test, and higher cytotoxicity than the e-cigarette. The metabolic activity of H292 cells decreased significantly after exposure to HTP emissions. At the same time, HTP emissions showed lower toxicity compared to flammable cigarettes, but higher toxicity compared to e-cigarettes. It was found that bronchial epithelial cells exposed to HTP emissions released less IL-1β and IL-6 than cells exposed to cigarette smoke [34].

Aerosol emitted from IQOS has been shown to damage human bronchial epithelial cells; however, the cytotoxicity of the IQOS was lower than that of the flammable cigarette, but showed a higher toxicity than the e-cigarette, which was in line with the tobacco industry data. The data suggest that the use of IQOS products may lead to an increased risk of respiratory disorders, and this risk is likely to be greater than the risk associated with e-cigarettes [34].

In a study by Walczak et al. (2020), the morphology and dynamics of the mitochondrial network in human bronchial epithelial cells (BEAS-2B) exposed to total particulate matter (TPM) generated by aerosol from a tobacco heating system (THS 2.2) was investigated. Comparison of the effects of TPM with 3R4F and THS 2.2 revealed that a similar range of changes in mitochondrial dynamics and biogenesis is observed at 7.5 µg/mL 3R4F TPM and 150 µg/mL THS 2.2 TPM. The seven-day exposure to the tested components of cigarette smoke causes mitochondrial stress, while the 12-week exposure showed signs of cellular adaptation to the stressor [36].

One independent study of the experience and behavioral aspects of using IQOS™ found positive short-term effects from overnight abstinence in humans. IQOS is as effective in reducing cigarette cravings and withdrawal symptoms as an e-cigarette, and is more preferred. Adriaens et al. (2018) showed that short term use of IQOS™ has minimal effect on exhaled CO (eCO). The use of IQOS resulted in a small but reliable increase in eCO (0.3 ppm) [43].

Only three studies conducted in humans showed that the use of heated tobacco products can cause negative health effects in humans, such as the occurrence of acute eosinophilic pneumonia (AEP) [40,41] and a negative impact on lung physiology [9].

Researchers from Japan reported two connections with eosinophilic capital, fuel after HTP subsidies [40,41]. The first case of acute eosinophilic supplementation (AEP is a rare disorder characterized by hypoxemia, pulmonary eosinophilia and pulmonary infiltrates) was diagnosed in a 20-year-old man who smoked 20 IQOS sticks a day for six months, and doubled his consumption of sticks two weeks before hospitalization. Chest X-ray showed bilateral opacities, and computed tomography of the chest showed smooth thickening of the interlobular septum, bilateral infiltrates and pleural effusion.

The white blood cell count was 15 690/mm^3^ (88% neutrophils, 7% lymphocytes and 1% eosinophilia), and the C-reactive search results were exceeded at the level of 10.12 IU/l mg/dl [40].

The second case of acute eosinophilic lung disease was diagnosed in a 16-year-old man with bronchial asthma who had been using HTP for two weeks. Symptoms of cough, shortness of breath and fatigue began immediately after smoking a heat-not-burn cigarette (HNBC), and worsened in the two weeks prior to hospitalisation. Blood tests showed an increase in C-reactive protein and leukocytosis (white blood cell count; neutrophils; lymphocytes; monocytes). The patient was diagnosed with AEP based on the clinical course and the detection of eosinophils in the sputum. The cytological results showed that the percentage of eosinophils, neutrophils and lymphocytes was 14.7%, 51.7% and 33.6%, respectively [41]. These are the first cases of AEP caused by smoking heat-not-burn cigarettes (HC) [40]; this may arise from the possibility that IQOS produces a lower particulate load to the lungs, such that neither inflammatory response nor sensitisation responses are suppressed.

Tabuchi et al. (2018) evaluate the potential effects of HTP on lung physiology. The study suggested that all people exposed to passive smoking, had at least one health symptom. The symptoms reported after being sacrificed to secondhand IQOS smoking were malaise, eye pain and a sore throat. The highest incidence of tobacco damage from secondary exposure to HTP smoke was observed among never-users of any tobacco products [9].

One study performed by Lee et al. (2019) found that the use of heated tobacco products is associated with asthma, allergic rhinitis and atopic dermatitis in adolescents. Initial diagnosis of atopic dermatitis may lead to a later systemic immune response. This study shows that it is important for adolescents to pay particular attention to smoking, including HTP and its relationship to allergy epidemics. The use of HTP itself was strongly associated with asthma, as was the dual use of cigarettes and HTP [42]. The results may indicate that adolescents use these products less heavily than adults. This data may support exposures to substances in HNB product aerosols that are sufficient to sensitise, and not sufficient to suppress the sensitisation.

In a study investigating the association between IQOS and the expression of the nasal platelet-activating factor receptor (PAFR), which influences bacterial adhesion that causes respiratory infection. It was observed that PAFR expression was significantly increased in nasal epithelial cells following IQOS exposure and adherence of pneumococci to nasal epithelial cells. This study provides preliminary evidence that the use of heated tobacco products increases susceptibility to respiratory infections and infection-induced asthma exacerbations [46].

In conclusion, the presented studies both in vitro or in humans suggest that there may be a positive correlation between the use of HTP and the occurrence of respiratory diseases (especially the negative impact on lung physiology, human bronchial epithelial cells, AEP, allergic rhinitis and asthma). Increase of the level of oxidative stress, mitochondrial dysfunction and increase infections in the respiratory track (Table 2).

#### 3.1.2. The Circulatory System

Most of the studies on the adverse effect of exposure to IQOS on the circulatory system assess the dependence of the supplied extract contained in the IQOS aerosol [2,47,48,49,50]. Three of these studies were performed in US [47,48,51] and subsequent ones in Japan [2] and Switzerland [49,50]. Most of these studies are in vitro studies, while only four were performed on humans [2,47,48,51].

In a study by Lopez et al. (2016), it was found that the low CO supply associated with the use of ECIG (electronic cigarettes) and LLTV (loose-leaf tobacco vaporiser) suggests that smokers who switch to these products may reduce the risk of cardiac dysfunction—CO-induced vascular disease. Moreover, LLTV significantly increased the concentration of nicotine in the plasma, and significantly reduced the severity of withdrawal symptoms in tobacco smokers [51]. Poussin et al. (2016) showed that IQOS aqueous aerosol extract reduced the effect on the adhesion of monocytic cells to coronary endothelial cells when treated with an aqueous reference smoke extract [49], which could potentially reduce the risk of cardiovascular disease compared to inflammatory diseases with cigarettes [50].

Comparisons at the level of genes and biological networks/pathways showed strong similarities in the molecular changes induced by high THS2 levels. Two aqueous extract concentrations compared to low concentrations of 3R4F aqueous extract in both cell types. The aqueous THS2.2 extract promoted the adhesion of monocytic cells to endothelial cells through similar mechanisms to that described for the 3R4F aqueous extract. These data indicate the potential of THS2.2 to reduce the risk of cardiovascular disease compared to smoking cigarettes [49].

A study by van der Toorn et al. (2015) showed that the extract prepared from the new MRTP (modified risk tobacco products) aerosol candidate was less cytotoxic, induced less inflammation and had a lower effect on chemotaxis compared to the smoke extract from the reference flammable cigarettes 3R4F [50].

A study by Lüdicke F. (2018) investigated several markers of cardiovascular risk and function, including fibrinogen, hs-CRP, homocysteine and blood pressure. The concentration of hs-CRP on day 90 was 6.4% lower in the mTHS group than in the mCC group, but it was 10.7% higher in the mTHS group than in the SA group [2]. Additionally, in the mTHS group, there was a reduction in 11-dehydro-thromboxane B2 (a biomarker of platelet activation), a soluble intracellular adhesion molecule-1 (biomarker of endothelial function) and 8-epi-prostaglandin F2α (a biomarker of oxidative stress), as well as an increase in high-density lipoprotein cholesterol (biomarker of lipid metabolism) compared to the mCC group [2].

In a study by Haziza et al. (2020), beneficial changes in lipid metabolism (total cholesterol and high and low-density cholesterol), endothelial dysfunction (soluble intercellular adhesion molecule-1), oxidative stress (8-epi-prostaglandin F2α) and cardiovascular risk factors (e.g., highly sensitive C-reactive protein) were observed in the mTHS group. The decreased exposure shown after switching to mTHS points to pathomechanics pathways underlying the development of smoking-related diseases, with some more pronounced effects in healthy-weight subjects. The data suggest that the reduced exposure shown after complete mTHS conversion may be associated with a positive effect on endothelial dysfunction and oxidative stress [47,48]. Ogden et al. (2015) noted that switching to tobacco heating cigarettes was associated with a reduction in the number of platelets sICAM-1 and WBC [52]. In a study by Biondi-Zoccai G et al. (2019), HNBC and EVC had less effect than TC on some dimensions of oxidative stress, antioxidant reserve, platelet function, and blood pressure. Moreover, HNBC had less effect on the soluble peptide derived from Nox2, 8-iso-PGF2α-III and vitamin E, and seemed more satisfying and able to reduce the urge to continue smoking than EVC [53].

The studies assessing the impact of exposure to HTP and cardiovascular outcomes are rare, but suggest the decreased risk of cardiovascular disease and decrease adhesion of monotic cell to coronary endothelial cell, 11-dehydro-thromboxane B2 (a biomarker of platelet activation), 8-epi-prostaglandin F2α (a biomarker of oxidative stress), total cholesterol, C-reactive protein, platelets and leukocytes. The most of the presented studies suggest the beneficial effect of change the cigarettes to IQOS, not the exposure to HNBC itself (Table 2).

### 3.2. Biomarkers of Exposure—A Link with Potential Health Effects

In addition, studies found that levels of exposure biomarker to HPHC (potentially harmful components) were significantly reduced in participants switching to heated tobacco products from conventional cigarettes (CC). This can reduce the risk of developing diseases related to smoking, e.g., on the part of the respiratory and circulatory systems.

In the study by Haziza et al. (2016), the levels of 15 biomarkers of exposure (COHb, S-PMA, MHBMA, 3-HPMA, total NNN, total NNAL, total 1-OHP, 4-ABP, 1-NA, 2-NA,—toluidine, CEMA, HEMA, 3-HMPMA and total 3-OH-B [a] P) were decreased in both THS and SA (abstinence group) groups compared to the baseline level in CC [54].

In a study by Haziza et al. (2020), switching from mCC to mTHS significantly reduced HPHC exposure to levels similar to those observed in people who abstained from smoking during the study period [47,48]. In a study by Lüdicke et al. (2016), switching to CHTP resulted in a significant reduction in all measured exposure biomarkers, including carboxyhemoglobin (by 43%), levels of monohydroxy-3-butenyl-mercapturic acid (MHBMA) compared to regular CC brand smokers [55]. In a study by Lüdicke et al. (2017), people who switched to THS 2.1 had a significant reduction in all exposure biomarkers compared to the CC group, including S-PMA (93%), 2-NA (89.1%) and MHBMA (88.5%) [56].

In the study by Lüdicke et al. (2018), the concentrations of carboxyhemoglobin, 3-hydroxypropyl mercapturic acid, monohydroxybutenylomer-capric acid and S-phenyl mercapturic acid were lower in the mTHS group than in the mCC group [57]. In a study by Gale et al. (2019), a reduction in exposure biomarkers (BoE) was observed in people switching to Glo, which was comparable to that observed after quitting smoking and to levels similar to those observed in non-smokers (reductions for NNAL, 3-HPMA, 4-ABP, HMPMA, eCO, MHBMA, 2-AN, S-PMA and CEMA) [58]. In people taking THS, a decrease in the enzymatic activity of CYP1A2 has also been shown, which indicates a reduced exposure to HPHC, which also reflects a favorable biological change associated with lower levels of active and carcinogenic metabolites [54,59].

Studies assessing the biomarkers of exposure to HTPs found a reduction of exposure in participants switching to heated tobacco products from conventional cigarettes (CC), which may suggest decrease risk of developing smoking-related diseases.

## 4. Discussion

Heated tobacco products should be subject to policies and regulatory measures that apply to all other tobacco products, in line with the WHO Framework Convention on Tobacco Control [60]. There is currently no evidence to show that HTPs are less harmful than conventional tobacco products [7,60]. According to the WHO, all forms of tobacco smoking are harmful, including heated tobacco products [60]. The European Respiratory Society (ERS) concludes that, as with normal smoking, heated tobacco products are addictive and carcinogenic to humans [61].

The studies assessing the impact of HTP on human health are rare. Most of them were conducted by the tobacco industry. However, the results of in vitro tests indicate that HTP aerosols have lower toxicity, and do not create new hazards compared to conventional cigarette smoke [62,63]. No sufficient number of longitudinal human studies are available to confirm that switching from conventional cigarettes to IQOS leads to a reduction in exposure to toxic fumes in a manner comparable to quitting smoking [2,57]. The reduction of exposure biomarkers may suggest the reduction of the risk of developing smoking-related diseases.

The studies that evaluated the effect of exposure to the respiratory system found that there may be a positive correlation between the use of HTP and the occurrence of respiratory diseases; particularly a negative impact on lung physiology, human bronchial epithelial cells, AEP, allergic rhinitis and asthma. Data from our review study suggests that exposure to IQOS contributes to altering mitochondrial function, which may further exaggerate airway inflammation, airway remodeling and lung cancer. These products have the potential to increase oxidative stress and increase respiratory tract infections by increasing microbial adherence to the respiratory tract.

On the other hand, studies assessing the impact of exposure to HTP and cardiovascular outcomes suggest a decreased risk of cardiovascular disease, as well as decreased adhesion of monotic cells to coronary endothelial cells, 11-dehydro-thromboxane B2 (a biomarker of platelet activation), 8-epi-prostaglandin F2α (a biomarker of oxidative stress), total cholesterol, C-reactive protein, platelets and leukocytes. The analysis of exposure biomarkers and cardiovascular biomarkers showed differences between smokers and people using heated tobacco products. Improvements in clinically relevant risk markers were observed compared to daily cigarette smokers.

Our study focuses on the health effects of smoking HTP products after they are introduced to the market for non-flammable products. Our review suggests that HTP products may be products with a reduced risk of chronic diseases, including respiratory and cardiovascular diseases and cancer, compared to traditional smoking, though in the case of non-smokers so far, they may pose a risk of their occurrence.

Currently, there is insufficient evidence to conclude that HTPs are less harmful than conventional cigarettes. Data on the health effects of heated tobacco products are limited. To date, single studies on the potential harm of HTP use have appeared in global literature. There are very few independent studies showing the short-term effects of HTP, and they indicate short-term physiopathological effects [34,35,53]. There is no evidence available to suggest that the use of HTP is associated with any long-term clinical outcomes related to exposure to the emission of these products.

Quitting smoking significantly reduces the risk of developing serious chronic diseases. It is now suggested and supported by tobacco sponsors that nicotine addicts should switch from regular smoking to alternative products that at least reduce the overall harm of smoking, though there are not a sufficient number of longitudinal studies to support such this idea. Nonetheless, there are less invasive and highly effective treatments (medications or behavioral interventions). On the other hand, heated tobacco products are another alternative that have appeared on the market alongside e-cigarettes. Studies have shown that current smokers with the intention to quit smoking used IQOS much more often than current smokers without the intention to quit smoking (18.8% vs. 10.3%) [9]. There is no evidence that HNB tobacco products such as IQOS currently play a role in smoking cessation [9].

Tobacco-related harm reduction (THR), by encouraging the substitution of low-risk alternatives, can be an alternative way to quit smoking, especially for those smokers who cannot or do not want to quit [43]. It was shown that five minutes of IQOS use resulted in a significant reduction in craving for cigarettes by 28%, while smoking reduced cravings by 44% [43].

However, HTP devices may not be a safer option compared to smoking or e-cigarette use, showing negative health effects for people who use them.

Studies have shown that e-cigarettes contain high levels of toxic compounds [64], which adversely affect the respiratory, gastrointestinal and cardiovascular systems both in vitro and in vivo [65,66]. It has been shown that the cardiovascular risk associated with the use of e-cigarettes is lower than the risk associated with traditional cigarette smoking, but may be a high risk for people with a predisposition to cardiovascular diseases [67].

People exposed to e-cigarette use may experience irritation of the upper respiratory tract and eyes, and the systemic effects of nicotine, including increased heart rate and higher systolic blood pressure [68]. HTP has been shown to emit more PAHs and carbonyls than is observed in e-cigarette fumes. HTP aerosol shows reduced cytotoxicity compared to cigarette smoke, but higher than that of e-cigarette fumes. Both HTP and e-cigarettes can trigger oxidative stress and an inflammatory response [69].

HTP can be much more harmful than e-cigarettes, including the emission of carbonyls (acrolein, acetaldehyde, formaldehyde) and PAHs [69].

Research into the long-term health effects of HTP products is limited, so it is difficult to say whether or not heated tobacco products will have adverse health effects in the future.

Human studies that measured plasma levels of nicotine after HTP use indicate that the delivery of nicotine by HTP varies from brand to brand, but is always lower than that provided by a conventional cigarette, except for IQOS [70].

Our review is one of the first to consider the short- and long-term health effects of using heated tobacco products on the human body. HTP users are adults, but mostly young adults.

Young people reaching for IQOS, Glo and Ploom, who are “following the fashion”, also want a “safe cigarette”. Presenting HTP as safer is another element that encourages young adults to try these products.

As research shows, the introduction of, among others, IQOS, will cause young non-users to start using tobacco with IQOS, and may also increase the use of IQOS with other tobacco products [71]. For conventional tobacco smokers, IQOS products can be an alternative option that helps reduce exposure to unsafe and potentially unsafe ingredients. However, non-smokers who use IQOS cigarettes may develop an addiction or increase exposure to certain substances, which may increase the likelihood of tobacco-related diseases [43]. The results of other studies in Europe, America and Great Britain show that HTP products will probably be very popular among teenagers due to the increased awareness of HTP products [11,13,72,73].

Additionally, a review by Ratajczyk et al. (2019) found that there is interest among young adults to try new HnB products, both current smokers and non-smokers, which raises concerns about new smokers. The susceptibility to trying IQOS (25.1%) was higher than that of traditional cigarettes (19.3%), but lower than that of e-cigarettes (29.1%). The process of popularising HnB tobacco products is global. People seem to believe that this product is more environmentally friendly, as it does not emit an unpleasant odor compared to traditional cigarettes [74]. In the review by Simonavicius et al. (2017), only three out of 31 studies were devoted to the impact of heated tobacco products on human health. HnB exposed users and bystanders to toxins; their concentrations were much lower than in the case of cigarettes. Research on passive HnB emissions and human use has been heterogeneous, and largely linked to tobacco producers [70].

In conclusion, there is lack of well-designed, longitudinal epidemiological studies on the health effects associated with IQOS exposure. Additionally, the differences or lack of the confounding factors were observed in the most of the presented studies. Human studies are based on a small sample size. Most of the presented studies are cross-sectional studies, where the exposure and effect is measured during the same period of time. The lack of detailed information about past cigarette smoking and past e-cigarette smoking showed that it is difficult to assess if the health effect is related only to IQOS smoking; the main problem is also evidence which indicates that in most countries where HTP are popular, they are unfortunately not used exclusively, and the data on health effects from exclusive use are irrelevant to the real-world use of these products.

## 5. Conclusions

The studies assessing the health effects of exposure to IQOS are rare, especially epidemiological studies. The presented studies, both in vitro or in humans, suggest that there may be a positive correlation between the use of HTP and the occurrence of respiratory diseases, particularly negative impacts on lung physiology, human bronchial epithelial cells, AEP, allergic rhinitis and asthma. Increase of the level of oxidative stress, mitochondrial dysfunction and increase infections in the respiratory track, decreased risk of cardiovascular disease and decreased adhesion of monotic cell to coronary endothelial cell, 11-dehydro-thromboxane B2 (a biomarker of platelet activation), 8-epi-prostaglandin F2α (a biomarker of oxidative stress), total cholesterol, C-reactive protein, platelets and leukocytes have been observed.

It seems necessary to undertake research to assess the frequency of HTP use and its potential impact on human health. Future research should focus on emissions of toxic and carcinogenic substances from heated tobacco products, including on the emission of carbonyls (acrolein, acetaldehyde and formaldehyde) and PAHs. Future studies should be designed as longitudinal studies, which will be able to measure the exposure in different windows of exposure. Future studies should be controlled for potential confounding factors. The bigger sample size may be achieved by performing international studies. The methodology, especially the definition of an IQOS smoker with additional information about past smoking, if it exists, should be clearly stated.

No tobacco product has been proven to be safe and risk-free. Research has shown that the benefits of HTP are controversial. More research is warranted to determine the short- and long-term health effects of using HTP products.

Governments should introduce a system for pre-market evaluation of novel tobacco products, including HTP.

Heated tobacco products can be an alternative to heavy smokers who have failed comprehensive treatment for nicotine addiction.

## Figures and Tables

**Table 1:** Studies included in the review.

Authors, Year of Publication	Material	Country	Funder	Main Objective
Van der Toorn, 2018	in vitro epithelial cells	Switzerland	dependent	functional and molecular changes in human bronchial epithelial BEAS-2B cells following a 12-week exposure to total particulate matter (TPM) from the aerosol of a candidate modified-risk tobacco product (cMRTP) in comparison with those following exposure to TPM from the 3R4F reference cigarette.
Sohal, 2019	in vitro human bronchial epithelial cells, human airway smooth muscle (ASM) cells	Australia	independent	exposure to IQOS has the same damaging effect on human airway epithelial and smooth muscle cells as traditional tobacco cigarette and eCigs in vitro.
Miyashita, 2018	human in vivo human airway epithelial cells	UK	independent	e-cigarette vapour increases pneumococcal adhesion to airway cells.
Malinska, 2018	in vitro human bronchial epithelial cells	Poland	dependent	assessment of mitochondrial function following short- and long-term exposure of human bronchial epithelial cells to total particulate matter from a candidate modified-risk tobacco product and reference cigarettes.
Leigh, 2018	in vitro human bronchial epithelial cells	US	independent	the potential toxic effects of inhaling emissions from an HTP in comparison with electronic and combustible tobacco cigarettes.
Walczak, 2020	in vitro human bronchial epithelial cells	Poland	dependent	morphology and dynamics of the mitochondrial network in human bronchial epithelial cells exposed to total particulate matter (TPM) generated from 3R4F reference cigarette smoke and from aerosol from a new candidate modified risk tobacco product, the Tobacco Heating System (THS 2.2).
Adriaens, 2018	human	Belgium	independent	to investigate the effect of using an IQOS™ on eCO, acute cigarette craving, withdrawal symptoms and subjective positive and negative experiences after overnight smoking abstinence, compared to using an e-cigarette or a regular tobacco cigarette; and to investigate which product (e-cigarette or IQOS™) would be preferred.
Kamada, 2016	human case report	Japan	independent	report the first case of AEP caused by smoking HC
Aokage, 2019	human case report	Japan	independent	report a successfully treated case of fatal AEP, presumably induced by HNBC use.
Tabuchi, 2018	human	Japan	independent	to assess interest in HnB tobacco products (including IQOS, Ploom and Glo), its prevalence in 2015, 2016 and 2017, to examine the symptoms from exposure to secondhand HNB tobacco aerosol in Japan.
Lee, 2019	human	Korea	independent	assessment of association of the HnB’s use with perceived stress, physical activity and internet use.
Lopez, 2016	human	US	independent	to expand existing clinical laboratory methods to examine, in cigarette smokers, the acute effects of a “heat, not burn” “loose-leaf tobacco vaporizer” (LLTV).
Poussin, 2016	in vitro human endothelial cells	Switzerland	dependent	to compare the biological impact of aqueous extracts from a candidate MRTP, Tobacco Heating System (THS) 2.2 (electrically-heated tobacco technology), and the 3R4F reference cigarette on monocytic cell-HCAEC adhesion combining functional measurements from an in vitro adhesion assay with transcriptomics and inflammatory protein marker data to investigate changes at the molecular level.
Van der Toorn, 2015	in vitro monocytic cell line and human coronary arterial endothelial cells	Switzerland	dependent	the effect from a new candidate modified risk tobacco product, the tobacco heating system (THS) 2.2, on the migratory behavior of monocytes in comparison with combustible 3R4F reference cigarettes.
Lüdicke, 2018	human	Japan	dependent	risk markers of smoking-related diseases.
Haziza, 2020a	human	US	dependent	the exposure reduction to selected HPHCs in smokers switching to menthol Tobacco Heating System (mTHS) 2.2 compared with smokers continuing smoking menthol cigarettes (mCCs) and smoking abstinence (SA).
Haziza, 2020b	human	US	dependent	offering an alternative to cigarettes for smokers while substantially reducing the exposure to harmful and potentially harmful constituents found in cigarette smoke.
Haziza, 2016a	human	Japan	dependent	demonstrate exposure reduction to a selected set of HPHCs when switching from CCs to THS 2.2, as compared to continued CC use and smoking abstinence (SA) for five days.
Lüdicke, 2016	human	Poland	dependent	to investigate the effects of exposure to selected harmful and potentially harmful constituents (HPHCs) of cigarette smoke in adult smokers who switched to a carbon-heated tobacco product (CHTP), compared with adult smokers who continued to smoke CCs and those who abstained from smoking for five days.
Lüdicke, 2017	human	Switzerland	dependent	examined whether the levels of selected biomarkers of exposure were reduced in smokers who switched from CCs to THS 2.1, as compared to smokers that continued to smoke CCs.
Lüdicke, 2018	human	Japan	dependent	examined the impact of switching to mTHS on biomarkers of exposure to HPHCs relative to menthol CCs (mCCs) and smoking abstinence (SA).
Ogden, 2015	human	US	dependent	evaluation of biomarkers of biological effect (e.g., inflammation, lipids, hypercoaguable state).
Biondi-Zoccai, 2019	human	Italy	independent	to appraise the acute effects of single use of HNBC, EVC and TC in healthy smokers.
Gale, 2021	human	UK	dependent	investigating whether biomarkers of exposure (BoE) to smoke toxicants are reduced when smokers switch from smoking cigarettes to using the glo THP.
Haziza, 2016b	human	Poland	dependent	to demonstrate a reduction in exposure to HPHCs.

**Table 2:** The adverse health effects of expsoure to IQOS/HTP.

Type of Outcome	Human Studies	In Vitro Studies
destructive effect on human respiratory epithelial cells/human bronchial epithelial cells	+ ↓ (Haziza, 2020)	+ ↓ (Van der Toorn, 2018) + ↑ (Leigh, 2018) + ↑ (Walczak, 2020) + ↑ (Malinska, 2018) + ↑ (Poussin, 2016) + ↑ (Miyashita, 2018) + ↑ (Sohal, 2019)
destructive effect on monocytic cell line and human coronary arterial endothelial cells	-	+ ↓ (Van der Toorn, 2018)
destructive effect on nasal epithelial cells	-	+ ↑ (Miyashita, 2018)
lung cancer risk	+ ↓ (Haziza, 2016a, 2016b) + ↓ (Lüdicke, 2016) + ↓ (Lüdicke, 2018)	+ ↓ (Van der Toorn, 2018)
AEP acute eosinophilic pneumonia	+ ↑ (Kamada, 2016) + ↑ (Aokage, 2019)	-
asthma, allergy, rhinitis	+ ↑ (Lee, 2019)	+ ↑ (Miyashita, 2018)
atopic dermatitis	+ ↑ (Lee, 2019)	-
oxidative stress/oxidative damage	+ ↑ (Ogden, 2015) + ↑ (Biondi Zoccai, 2019) + ↓ (Haziza, 2020)	+ ↑ (Sohal, 2019) + ↑ (Malinska, 2018)
inflammation, infections in the respiratory tract	-	+ ↑ (Sohal, 2019) + ↑ (Miyashita, 2018) + ↓ (Van der Toorn, 2016)
mitochondrial dysfunction/mitochondrial stress	-	+ ↑ (Malinska, 2018) + ↑↓ adaptation (Walczak, 2020)
the risk of cardiovascular disease	+ ↓ (Lopez, 2016) + ↓ (Lüdicke, 2018)	+ ↓ (Poussin, 2016) + ↓ (Van der Toorn, 2016)
atherosclerosis	+ ↑ (Biondi Zoccai, 2019)	-
adhesion of monocytic cells to coronary endothelial cells	-	+ ↓ (Poussin, 2016) + ↓ (Van der Toorn, 2016)
11-dehydro-thromboxane B2 (a biomarker of platelet activation)	+ ↓ (Haziza, 2020) + ↓ (Gale, 2021)	-
adhesion molecule-1 (biomarker of endothelial function	+ ↓ (Lüdicke, 2018)	-
8-epi-prostaglandin F2α (a biomarker of oxidative stress)	+ ↓ (Lüdicke, 2018) + ↓ (Haziza, 2020)	-
high-density lipoprotein cholesterol	+ ↑ (Lüdicke, 2018) + ↓ (Haziza, 2020)	-
low-density lipoprotein cholesterol	+ ↑ (Haziza, 2020)	-
total cholesterol	+ ↓ (Haziza, 2020)	-
C -reactive protein	+ ↓ (Haziza, 2020)	-
Intracellural adhesion molecule 1	+ ↓ (Haziza, 2020)	-
Blood morphology: palatelets, leukocytes	+ ↓(Ogden, 2015)	-
exposure biomarkers (COHb, S-PMA, MHBMA, 3-HPMA, total NNN, total NNAL, 1-OHP, 4-ABP, 1-NA, 2-NA, o-tol, CEMA; HEMA, 3-HMPMA i total 3-OH-B [a] P, carbon monoxide, benzene, 1–3 butadiene, acrolein, eCO, nicotine exposure (plasma nicotine, cotinine, TNeq))	+ ↓ (Haziza, 2016) + ↓ (Haziza, 2020) + ↓ (Lüdicke, 2016) + ↓ (Lüdicke, 2017) + ↓ (Lüdicke, 2018) + ↓ (Ogden, 2015) + ↓ (Gale, 2021)	-
reducing cigarette cravings and withdrawal symptoms	+ ↓ (Adriaens, 2018) + ↓ (Lopez, 2016)	-

+ effect (↓ decrease of the effect, ↑ increase of the effect). - no effect.

## Abbreviations/Acronyms

HTP	heated tobacco products
sICAM-1	soluble intercellular adhesion molecule 1
8-epi-PGF2α	8-epi-prostaglandin F2α
11-DTX-B2	11-dehydro-thromboxane B2
HDL	high density lipoprotein
FEV_1_	forced expiratory volume in 1 s
IQOS	I-Quit-Ordinary-Smoking
PMI	Philip Morris International
JTI	Japan Tobacco International
BAT	British American Tobacco
HNB, HnB	heat-non-burn
THS 2.2	The Tobacco Heating System 2.2
CC	Smoking conventional cigarettes
HPHC	the harmful and potentially harmful constituents
COHb	Carboxyhemoglobin
S-PMA	S-phenylmercapturic acid
MHBMA	Monohydroxybutenyl mercapturic acid
3-HPMA	3-hydroxypropylmercapturic acid
NNN	N-nitrosonornicotine
Total NNN	Total N-nitrosonornicotine
Total NNAL	Total 4-(methylnitrosamino)-1-(3-pyridyl)-1-butanol
o-tol	o-toluidine
1-OHP	Total 1-hydroxypyrene
4-ABP	4-aminobiphenyl
1-NA	1-aminonaphthalene
2-NA	2-aminonaphthalene
CEMA	2-cyanoethylmercapturic acid
HEMA	2-hydroxyethylmercapturic acid
3-HMPMA	3-hydroxy-1-methylpropylmercapturic acid
Total 3-OH-B [a] P 3	hydroxy-benzo(a)pyrene
SA	smoking abstinence group
CYP1A2	Cytochrome 1 A2 activity
CHTP	carbon-heated tobacco product
mTHS	The menthol Tobacco Heating System 2.2
mCC	menthol cigarettes
WBC	white blood cells
hs-CRP	high-sensitivity C-reactive protein
THS 2.1	The Tobacco Heating System 2.1
CO	carbon monoxide
HNBC	heat-not-burn cigarette
EVC	electronic vaping cigarettes
TC	traditional tobacco combustion cigarettes
Nox2 soluble Nox2	derived peptide
8-izo-PGF2α-III	8-iso-prostaglandin F-2α-III, pmol/L
BoE	biomarkers of exposure
NNAL	4-(methylnitrosamino)-1-(3-pyridyl)-1-butanol
HMPMA	eCO exhaled breath carbon monoxide
MHBMA	monohydroxy-3-butenyl-mercapturic acid
H292	human bronchial epithelial cells
IL-1β	interleukin (IL)-1β
IL-6	interleukin (IL)- 6
BOPH	biomarkers of potential harm
TPM	total particulate matter
3R4F	reference cigarette smoke
PAFR	platelet-activating factor receptor
CSE	cigarette-smoke-extract
ASM	airway smooth muscle
EMT	epithelial mesenchymal transition
BEAS-2B	human bronchial epithelial cells
CS	cigarette smoke
AEP	acute eosinophilic pneumonia
HNBC	heat-not-burn cigarette
ECMO	extracorporeal membrane oxygenation
HC	heat-not-burn cigarettes
ECIG	electronic cigarettes
LLTV	loose-leaf tobacco vaporiser
MRTP	modified risk tobacco products
THR	Tobacco Harm Reduction
TNeq	total nicotine equivalents (nicotine, cotinine, 3-hydroxycotinine and their glucuronide conjugates)
PAHs	Polycyclic aromatic hydrocarbons
